# Phenotype, Squalene, and Lanosterol Content Variation Patterns During Seed Maturation in Different Leaf-Color Tea Cultivars

**DOI:** 10.3390/foods15010094

**Published:** 2025-12-29

**Authors:** Jing-Jing Ye, Yu-Ning Fang, Xiao-Quan Lu, Shu-Ling Dong, Yue-Rong Liang, Jian-Liang Lu, Kai-Rong Wang, Long-Jie Zhang, Xin-Qiang Zheng

**Affiliations:** 1 Tea Research Institute, Zhejiang University, Hangzhou 310058, China; 2 General Agrotechnical Extension Station of Ningbo City, Ningbo 315000, China; 3 Ningbo Huangjinyun Tea Science and Technology Co., Ltd., Ningbo 315412, China

**Keywords:** leaf-color specific cultivars, maturity, seed, squalene, lanosterol

## Abstract

Squalene and lanosterol are bioactive compounds with diverse physiological effects, found in relatively high concentrations in tea seed oil. Their levels are significantly influenced by cultivar and fruit maturity. As leaf-color specific tea cultivars gain popularity, parts of them tend to have a higher flower and fruit ratio than green-leaf tea cultivars. However, their fruit characteristics remain underexplored. This study investigated 15 tea cultivars with different leaf colors, analyzing phenotypic changes in seeds during maturation, and examining the variation patterns of squalene and lanosterol. The crude water content, dry kernel content, and oil content were closely related to the maturity and effectively reflected seed development. Lanosterol content showed an overall downward trend with increased maturity. Squalene content fluctuated sharply before the seeds fully matured, but gradually decreased once they were fully matured. At full maturity, leaf-color specific tea cultivars generally exhibited higher concentrations of squalene and lanosterol than those with green leaf.

## 1. Introduction

Natural products are predominantly endogenous chemical substances and secondary metabolites synthesized by animals, plants, and microorganisms, which serve as rich sources for the development of functional foods and contemporary pharmaceuticals [1]. Among them, squalene and lanosterol are metabolic products in the biosynthesis pathway of plant terpenoid compounds. And the latter is a downstream product of the former. Existing reports have found that squalene has various physiological functions, including the prevention of cardiovascular diseases, anti-tumor, and antioxidant properties [2,3,4,5], while lanosterol contributes to the treatment of cataracts, hypertension, and Alzheimer’s disease, and can be used for radiation protection and immune enhancement [6,7,8,9]. These compounds are widely applied in food, pharmaceuticals, cosmetics, and other fields [10,11,12]. In plants, squalene accumulates significantly under drought, light, and temperature stress, indicating its potential role in enhancing plant stress resistance [13,14]. A study has shown that squalene is an important feedback signal that enhances the resistance of tea plants to cold by upregulating the expression of *CsCBF5* [15]. Furthermore, squalene may play an antimicrobial role during the occurrence of leaf spot disease in sesame (*Sesamum indicum* L.) [16]. The resistant citrus species have a higher ratio of free sterols (containing lanosterol) to total phospholipids than susceptible species [17]. The deletion of lanosterol synthase gene *Las1* reduced the ergosterol content and impaired cell membrane integrity [18].

Shark liver oil was once the richest natural source of squalene [19], but with the increasing demand for squalene, the limitations of animal-derived squalene sources have become more prominent. Lanosterol is also primarily derived from animal material extraction. Plant sources are more abundant and accessible than animal sources, with great potential for development. Therefore, the development and utilization of plant-derived bioactive ingredients are crucial for addressing resource scarcity, and identifying plant materials with high content and exploring their accumulation patterns are key. In plants, squalene is mainly found in seeds, oils, and leaves [20], while lanosterol is mostly detected in oils [21]. In traditional oil crops, squalene has been found in certain amounts in olive oil, *Amaranthus* seed oil, *Camellia oleifera* seed oil, rapeseed oil, sesame oil, and palm oil, etc. [22,23,24,25,26,27,28], with olive oil being the earliest and most commonly used commercial source of squalene. Non-traditional plant oils (e.g., from fruits, vegetables, herbs) also contain squalene, including rambutan seed oil, mango kernel oil, peony seed oil, gardenia fruit oil, linden seed oil, oat oil, Brazil nut oil, pumpkin oil, and *Kadsura coccinea* seed oil [29,30,31,32,33,34,35,36,37]. In addition, lanosterol has been found in refined olive oil, rice bran oil, rice oil, peanut oil, sesame oil, soybean oil, rapeseed oil, and tomato seed oil, while it has not been detected in palm oil, sunflower seed oil, and corn oil [21,38].

Tea plants (*Camellia sinensis* (L.) O. Kuntze) are an evergreen shrub or small tree belonging to the *Camellia* genus of the *Theaceae* family. In recent years, researchers have been studying the comprehensive utilization of tea seed resources, with oil extraction being the most common utilization method [39]. As a novel plant oil, tea seed oil is rich in various bioactive components, with a relatively high content of squalene and lanosterol [40]. However, research and the application of tea seed oil are still lacking compared to conventional woody oil crops (such as *C. oleifera* seed oil and olive oil). Current studies on these two components in tea plants remain relatively limited. Most existing research primarily compared their content variations in tea seed oils from different geographical origins, while with inconsistent conclusions [41,42,43,44]. Furthermore, investigations have predominantly focused on green-leaf cultivars, while the impact of varietal factors on component accumulation requires further exploration [45]. The oil yield of tea seeds directly affects the squalene and lanosterol content, making it crucial to select high-yield cultivars. Currently, tea germplasm with various specific leaf colors such as purple–black, purple, orange, red, yellow, white, green, and multicolored have been successfully cultivated [46]. Field trials also reveal that leaf-color specific cultivars, such as ‘Ziyazhong’ and ‘Ruixue 1 Hao’, exhibit significantly higher floral bud initiation rates and fruit set efficiency. These unique resources not only differ from green cultivars in appearance, but also exhibit distinct biochemical components, which may become important sources for squalene and lanosterol production. However, compared to green-leaf cultivars, the studies on the squalene and lanosterol content in tea seeds of these cultivars and the accumulation patterns with maturity are still scarce. Existing studies indicate that maturity, planting region, and cultivars are important factors affecting the content of squalene and lanosterol [47,48,49]. The influence of maturity and cultivars on quality has also been observed in studies on olive and camellia oils [50,51,52]. Therefore, it is necessary to clarify the accumulation patterns of squalene and lanosterol in tea seeds of different leaf-color cultivars during maturation to effectively improve resource utilization. It is believed that the development and application of tea seed oil help enhance the added value of products and provide consumers with more diversified, higher-quality health food choices.

This study observed the developmental stages of tea seeds from 15 different leaf-color tea cultivars, measured various seed traits, and used gas chromatography–mass spectrometry (GC-MS) to determine the squalene and lanosterol content in tea seed oil. The aims are to figure out the optimal harvesting maturity and seed traits for high bioactive components (squalene, lanosterol), and screen candidate cultivars for oil, squalene, and lanosterol utilization.

## 2. Materials and Methods

### 2.1. Materials

Tea fruits were collected from 15 tea plant cultivars with different leaf colors starting on 16 September 2022. In other reported studies [53,54,55,56,57], the interval between sampling events ranged from 7 to 20 days. Considering the maturation cycle of tea fruits, we ultimately determined a 7-day sampling interval for the study. The cultivars included yellow-leaf cultivars ‘Huangjinya’ (HJY), ‘Huangjinjia’ (HJJ), and ‘Yuehuang 1 Hao’ (YH1); purple-leaf cultivars ‘Ziyazhong’ (ZY) and ‘Zijuan’ (ZJ), white-leaf cultivars ‘Baiye 1 Hao’ (BY1), ‘Ruixue 1 Hao’ (RX1), and ‘Shuxue’ (SX); variegated-leaf cultivar ‘Jinyumantang’ (JYMT); and green-leaf cultivars ‘Longjing 43’ (LJ43), ‘Zhongcha 108’ (ZC108), ‘Zhenong 117’ (ZN117), ‘Jinxuan’ (JX), ‘Yingshuang’ (YS), and ‘Longjing Changye’ (LJCY). All samples were harvested in Zhejiang Province.

Most existing studies distinguish fruit maturity based on harvesting time and pericarp morphology [53,57,58], while the sampling in this study was mainly performed according to the time course. However, the developmental stages of tea fruits varied during growth; therefore, pericarp morphology was also taken into consideration as a reference. Specifically, at each sampling time point, we randomly collected samples from 50 tea plants of each cultivar. Finally, only those tea fruits with consistent traits (pericarp color and texture), accounting for more than 80% of the total fruits at the corresponding developmental stage, were selected for collection, ensuring that the fresh weight of each sample was approximately 300 g. For each sample, 50 g fruits were excised, flash-frozen in liquid nitrogen, and stored at −80 °C. In total, 250 g of fruits was manually separated into pericarp and seed coat, photographed, and subsequently used for seed trait measurements and compositional analysis. This indicated that each biological replicate was approximately derived from 80 g of tea fruits.

### 2.2. Seed Traits Measurements

#### 2.2.1. Seed Kernel-Related Traits

For each sample, a subset of tea fruits was randomly selected. The initial fruit weight (*m*_0_), number of healthy seeds (*x*_1_), and their weight (*m*_1_) were recorded. After removing the seed coat, the number of intact kernels (*x*_2_) and their weight (*m*_2_) were measured. The kernels were then dried at 80 °C in DHG-9240A Electrically Heated Blast Drying Oven (Shanghai Yiheng Scientific Instruments Co., Ltd., Shanghai, China), with hourly weighings after 8 h until the difference between consecutive measurements stabilized within 0.1 g. The final weight (*m*_3_) was recorded after cooling to room temperature. Three biological replicates were set for each sample.

Fresh kernel yield of fresh fruit (KF, %) = $\frac{m_{2}}{m_{0}}$ × 100

Fresh kernel yield of fresh seed (KS, %) = $\frac{m_{2}}{m_{1}}$ × 100

Crude water content of kernels (CWC, %) = $\frac{m_{2}-m_{3}}{m_{2}}$ × 100

Dry kernel yield of fresh fruit (DKF, %) = $\frac{m_{3}}{m_{0}}$ × 100

Dry kernel yield of fresh seed (DKS, %) = $\frac{m_{3}}{m_{1}}$ × 100

#### 2.2.2. Oil Content of Dry Kernels

Dry kernels were ground and sieved through a 40-mesh screen. Crude fat was extracted and measured using the Soxhlet method following GB/T 14488.1-2008 [59]. The equipment was SOX406 Soxhlet Extractor (Haineng Future Technology Group Co., Ltd., Jinan, China).

Approximately 3.00 ± 0.10 g of powdered dry kernels (*m*_4_) was wrapped in defatted cotton and secured in filter paper. The extraction flask was dried to a constant weight (*m*_5_), then filled with 50 mL petroleum ether (boiling range: 30–60 °C, Sinopharm Chemical Reagent Co., Ltd., Shanghai, China), and the sample was fully immersed for 2 h, followed by 1 h of rinsing and 30 min of solvent recovery. The flask was then dried to a constant weight (*m*_6_). Three biological replicates were set for each sample. The oil was stored at −20 °C for further analysis.

Oil content of dry kernels (ODK, %) = $\frac{m_{6}{-m}_{5}}{m_{4}}$ × 100

### 2.3. Analysis of Squalene and Lanosterol Content

#### 2.3.1. Saponification and Unsaponifiable Matter Extraction of Tea Seed Oil

Following the method of Shi et al. [21], 5α-cholestane (purity ≥ 97%, Shanghai Aladdin Biochemical Technology Co., Ltd., Shanghai, China) was used as an internal standard (IS). A 250 µg/mL IS solution was prepared by dissolving 5α-cholestane in n-hexane (purity ≥ 97%, Sinopharm Chemical Reagent Co., Ltd., Shanghai, China). Squalene standard (purity ≥ 98%, Sigma-Aldrich Biochemical Technology Co., Ltd., Wuxi, China) and lanosterol standard (purity ≥ 99%, Sigma-Aldrich Biochemical Technology Co., Ltd., Wuxi, China) were similarly dissolved in n-hexane to prepare calibration solutions.

Approximately 200.00 ± 10.00 mg of tea seed oil (*m*_7_) was weighed, mixed with 200 µL IS solution and 0.5 mL saponification solution (2 M KOH dissolved in ethanol), and incubated in 85 °C for 1 h, followed by rapid cooling to room temperature. Subsequently, 0.5 mL water and 0.5 mL n-hexane were added, vortexed thoroughly, and centrifuged at 4500 rpm for 10 min. The upper organic phase was collected for analysis. Three biological replicates were set for each sample.

#### 2.3.2. GC-MS Analysis of Squalene and Lanosterol in Tea Seed Oil

Following the method of Sheng et al. [60] and combining with other studies [53,54,61,62], squalene and lanosterol content were determined using a QP2010 Ultra GC-MS system equipped with an AOC-20i autosampler (Shimadzu Corporation, Kyoto, Japan) and a DB-5 capillary column (30 m × 0.25 mm × 0.25 µm, Agilent Technologies, Inc. Santa Clara, CA, USA). High-purity helium (99.999%) served as the carrier gas. Instrumental parameters were as follows: pre-injection solvent wash for 2 cycles, post-injection solvent wash for 3 cycles, sample wash for 2 cycles, viscosity delay of 0.2 s, and injection volume of 1 µL.

For the GC unit, injector temperature 315 °C, split ratio 20:1, flow control mode 130.2 kPa, total flow 24.0 mL/min, column flow 1.0 mL/min, purge flow 3.0 mL/min. Oven program, initial 250 °C (hold 0 min), ramp 7.5 °C/min to 286 °C, final hold 18 min.

For the MS unit, ion source temperature 230 °C, interface temperature 250 °C, solvent delay 2 min, detector voltage 1.2 kV, and scan range 30–500 *m*/*z*.

#### 2.3.3. Squalene and Lanosterol Identification and Quantification

Squalene and lanosterol were identified by comparing retention times with the standard and cross-referencing the NIST GC-MS database. Quantification followed the internal standard method [43], using the peak area ratio of squalene to 5α-cholestane (IS). The formula was as follows:

$$W_{1}=\frac{S_{1}\times C\times V}{S_{c}\times m_{7}}$$

$$W_{2}=\frac{S_{2}\times C\times V}{S_{c}\times m_{7}}$$

*W*_1_, squalene content in tea seed oil (mg/kg); *W*_2_, lanosterol content in tea seed oil (mg/kg); *S*_1_, peak area of squalene; *S*_2_, peak area of lanosterol; *S*_c_, peak area of 5α-cholestane (IS); *C*, IS solution concentration (µg/mL); *V*, volume of IS solution added (µL); *m*_7_, weight of tea seed oil sample (mg).

### 2.4. Data Analysis and Visualization

Data processing was performed using Excel 2019, and statistical significance was assessed with SPSS 26.0. The data were statistically analyzed by one-way analysis of variance (ANOVA). Mean values between different samples were compared by Turkey’s multiple tests, and significant differences were established at *p* < 0.05. Figures and principal component analysis (PCA) were generated using Origin 2024. The heatmap was visualized by TBtools v1.098 [63].

## 3. Results

Overall, except for LJ43, ZN117, YS, and LJCY (sampling began on 30 September), all other cultivars were sampled starting on 16 September, with varying termination dates. BY1 and SX were the earliest to complete sampling, while LJ43 and JX were the latest. JX had the longest sampling duration, covering nine time points. Finally, a total of 90 samples were collected. The differences in fruit development and maturation periods may be attributed to genetic factors, climate, soil conditions, and agronomic practices.

**Figure 1:**
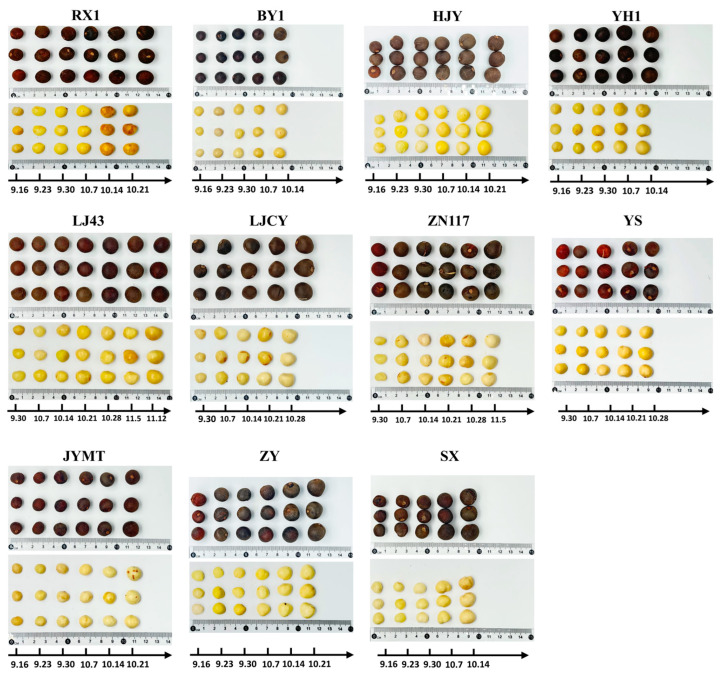
Phenotypes of tea seeds and kernels of some cultivars harvested in the maturation process. The same column represented the state of the tea seeds/tea seed kernels after peeling off the peels of tea fruits of the same cultivars harvested on the same day, which had a similarity in traits (the color and texture of peel) of more than 80% of all tea fruits that developed during the same period of time.

As shown in Figure 1 (only part of the images was displayed), tea seeds and kernels exhibited a spherical shape. However, varietal differences were observed in appearance and size. The seeds of HJY displayed a yellowish-brown hue, while RX1 and YH1 exhibited a dark brown coloration. The kernels of BY1 and JYMT remained relatively small, with minimal diameter increase during maturation. SX and LJCY showed significant diameter expansion as maturity progressed. YS and ZN117 consistently had larger seed diameters (similar trends were observed for kernel).

### 3.1. Crude Water Content and Dehydration Rate During Maturation

**Figure 2:**
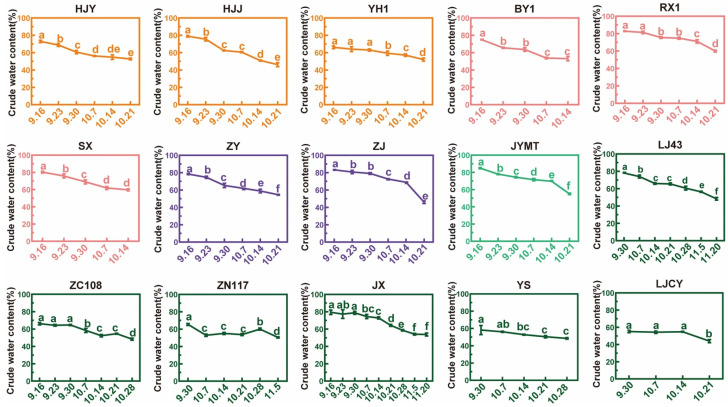
Changes in crude water content of kernels in tea seeds harvested in maturation process. The horizontal axis represented the picking date of samples. ‘Huangjinya’ (HJY), ‘Huangjinjia’ (HJJ), and ‘Yuehuang 1 Hao’ (YH1) were yellow-leaf cultivars, marked in orange; ‘Baiye 1 Hao’ (BY1), ‘Ruixue 1 Hao’ (RX1), and ‘Shuxue’ (SX) were white-leaf cultivars, marked in pink; ‘Ziyazhong’ (ZY) and ‘Zijuan’ (ZJ) were purple-leaf cultivars, marked in purple; ‘Jinyumantang’ (JYMT) was variegated-leaf cultivar, marked in cyan; ‘Longjing 43’ (LJ43), ‘Zhongcha 108’ (ZC108), ‘Zhenong 117’ (ZN117), ‘Jinxuan’ (JX), ‘Yingshuang’ (YS), and ‘Longjing Changye’ (LJCY) were green-leaf cultivars, marked in green. Same color coding for all following figures. Different letters above bars indicated significant differences using Duncan’s tests (*p* < 0.05).

The CWC during maturation was presented in Figure 2. Differences were observed among cultivars at both initial and final sampling stages. At the initial stage, the CWC of ZJ, RX1, SX, and JYMT exhibited higher (>80%) moisture, while YS and LJCY had <60% moisture. At the final stage, all seeds were nearly mature. RX1 retained the highest moisture (60.01%), while LJCY had the lowest (43.97%). Overall, the leaf-color specific cultivars generally maintained higher moisture levels.

The process of fruit ripening is a process of organic compound synthesis and accumulation, characterized by vigorous metabolism, which requires respiration to provide energy. Water is an important influencing factor in respiration. The CWC of the 15 cultivars generally showed a significant declining trend during the maturation process. The dehydration rate (calculated as moisture loss per day) between the initial and final harvesting points varied among cultivars. ZJ exhibited the highest rate at 1.066%/d, followed by HJJ at 0.936%/d, JYMT at 0.853%/d, BY1 at 0.782%/d, SX at 0.735%/d, LJ43 at 0.708%/d, ZY at 0.680%/d, RX1 at 0.651%/d, HJY at 0.578%/d, LJCY at 0.530%/d, JX at 0.457%/d, ZN117 at 0.427%/d, ZC108 at 0.422%/d, YH1 at 0.401%/d, and YS with the lowest rate at only 0.343%/d, approximately one-third of ZJ. The average water loss rate across all cultivars was 0.638%/d. ZJ remained relatively stable at below 0.923%/d in the early stages but exhibited the fastest rate (3.290%/d) from 14 October to 21 October. In contrast, YS had a water loss rate of only 0.343%/d during the same period, with rates consistently below 0.470%/d across all phases. So, differences were exhibited between ZJ and YS at every maturation stage.

For nine cultivars (HJJ, HJY, YH1, ZJ, ZY, RX1, JYMT, ZC108, and JX) all sampled at six time points (16 September–21 October), the average dehydration rate was stable in the first four maturation phases, which were 0.441%/d, 0.650%/d, 0.540%/d, and 0.524%/d. And a sharp decrease appeared in the last phase (14–21 October), namely 1.135%/d, which was consistent with ZJ’s trend.

### 3.2. Dry Kernel Content and Its Rate of Change in Fresh Fruits and Seeds During Maturation

During the maturation process, the KF and KS (here referring to the fresh kernel content) of each cultivar exhibited irregular fluctuations, even showing negative trends, with HJY being the most pronounced example (Table 1). No significant differences were observed in either KF or KS, which may be related to the excessively rapid water loss rate of tea seed kernels during maturation. Although dry matter continuously accumulates, excessive water loss may lead to negative fluctuations in fresh kernel yield. This suggested that changes in KF and KS during tea seed maturation were inconsistent.

**Figure 3:**
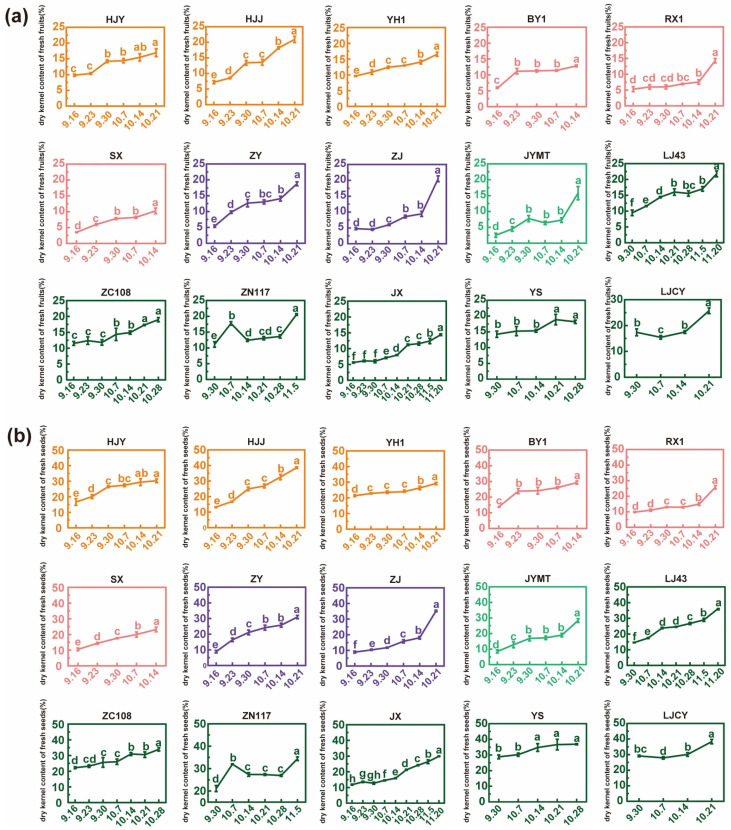
Changes in (**a**) dry kernel yield of fresh tea fruits (DKF) and (**b**) dry kernel yield of fresh tea seeds (DKS) harvested in maturation process. Different letters above bars indicated significant differences using Duncan’s tests (*p* < 0.05).

In production, tea seed kernels were typically dried before further processing, and the DKF and DKS were more useful for screening oil-use cultivars. Therefore, subsequent analyses focused directly on them (Figure 3a,b). At the initial harvest point, ZC108, ZN117, YS, and LJCY exhibited relatively high DKF (>10%) and DKS (>25%). In contrast, SX and JYMT had low DKF (<4%), while ZJ, ZY, and JYMT showed low DKS (<9%). At the final harvest point, HJJ, ZJ, LJ43, ZN117, and LJCY had high DKF (>20%), whereas HJJ, LJ43, YS, and LJCY displayed high DKS (>36%). Conversely, SX, BY1, RX1, and JX had low DKF (<15%), and SX showed particularly low DKS (<25%). Overall, among the 15 cultivars, all green-leaf cultivars (except JX) exhibited high dry matter content, with YS and LJCY maintaining consistently high levels throughout development. In contrast, the specific leaf-color cultivars ZJ and HJJ demonstrated high dry matter content only in the later stages of maturation.

The DKF and DKS exhibited a generally significant increasing trend across all cultivars during maturation. Minor fluctuations observed may be attributed to systematic errors in field experiments. The dry matter accumulation rate of kernels was calculated as the difference in DKF between the final and initial harvest points divided by the number of days (d). Varietal differences were observed. ZJ had the highest rate (0.450%/d), followed by LJCY (0.397%/d), HJJ (0.392%/d), ZY (0.382%/d), JYMT (0.379%/d), LJ43 (0.293%/d), ZN117 (0.271%/d), RX1 (0.257%/d), BY1 (0.253%/d), SX (0.241%/d), HJY (0.203%/d), YH1 (0.198%/d), ZC108 (0.178%/d), JX (0.157%/d), and YS (0.140%/d, approximately one-third of ZJ). The average dry matter accumulation rate across all cultivars was 0.280%/d. Analysis of the nine selected cultivars (mentioned in Section 3.1) revealed stable dry matter accumulation rates in the first four maturation phases (0.180%/d, 0.274%/d, 0.113%/d, and 0.182%/d). However, the final phase (14–21 October) showed the fastest (0.689%/d), coinciding with the highest water loss rate during the same period.

### 3.3. Oil Content and Its Rate of Change in Dry Kernels During Maturation

**Figure 4:**
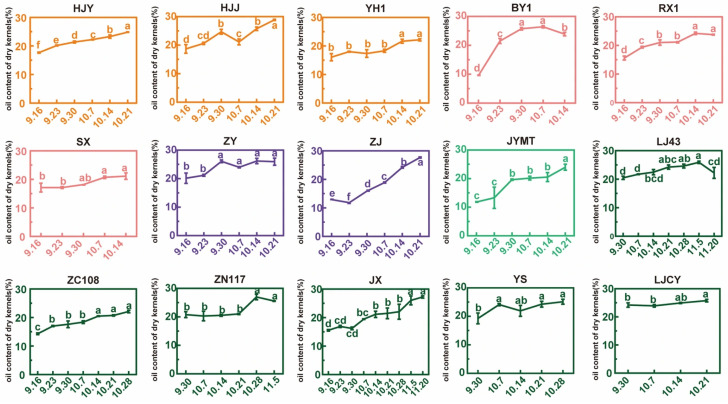
Changes in oil content of dry kernels obtained in maturation process. Different letters above bars indicated significant differences using Duncan’s tests (*p* < 0.05).

The trends in ODK across different cultivars during maturation were shown in Figure 4. Most exhibited a gradual increase, though some (ZY, BY1, RX1, LJ43, and ZN117) showed a slight decline in later stages. Minor fluctuations during maturation may also reflect environmental influences. At the initial harvest point, BY1 had the lowest ODK (9.57%), while ZY, LJ43, ZN117, and LJCY showed higher levels (>20%). By the final harvest, HJJ exhibited the highest ODK (28.88%), followed by ZJ and JX (>26%), whereas SX (21.13%), ZC108 (22.11%), and YH1 (22.15%) were relatively lower.

The lipid accumulation rate was calculated as the difference in ODK between the final and initial harvest points divided by the number of days. Differences were observed among cultivars. BY1 had the highest lipid accumulation rate (0.511%/d), followed by ZJ (0.422%/d), JYMT (0.348%/d), HJJ (0.291%/d), RX1 (0.233%/d), JX (0.212%/d), YS (0.209%/d), HJY (0.204%/d), ZC108 (0.186%/d), YH1 (0.174%/d), ZY (0.166%/d), SX (0.143%/d), ZN117 (0.137%/d), LJCY (0.075%/d), and LJ43 (0.041%/d). Notably, the accumulation rate in BY1 was approximately 6.8 times that of LJ43, primarily due to its initially low oil content, though their final levels were comparable. The average accumulation rate across all cultivars was 0.223%/d. For the nine selected cultivars (mentioned in Section 3.1), the lipid accumulation rates across maturation cycles were 0.248%/d, 0.341%/d, 0.061%/d, 0.377%/d, and 0.190%/d during 16 September–21 October, peaking during 7–14 October. This pattern differed from trends in other measured traits.

### 3.4. Comprehensive Analysis of Tea Seed Traits During Maturation

The four traits—CWC, DKF, DKS, and ODK—exhibited consistent directional changes (decrease/increase) across maturation stages. Prior studies confirmed that tissue maturation drove shifts in CWC, dry matter, and oil content [45,56,64]. Thus, we graded maturation levels based on harvest time; the initial sampling date was designated as maturity level 1, with increments for each subsequent interval (e.g., HJY samples from 9.16, 9.23, 9.30, 10.7, 10.14, and 10.21 were assigned levels 1–6).

Pearson’s correlation analysis revealed significant associations between maturation levels and all four traits (Table 2), confirming that four traits were reliable proxies for maturation. This supported the validity of using harvest time as a maturity indicator under our experimental conditions.

**Figure 5:**
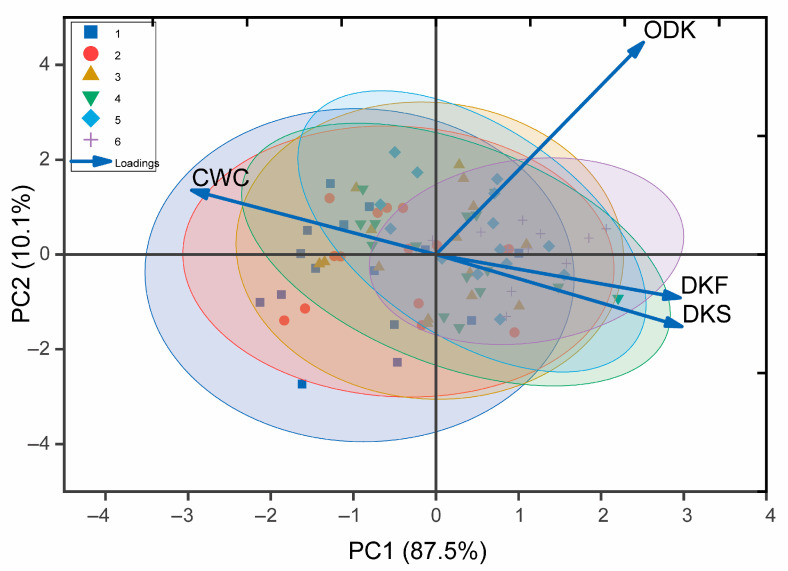
The loadings plot of PCA performed on maturity and cultivars.

The PCA analysis was conducted on the four seed traits of the 85 samples, using the maturity levels described above (1–6, with only five samples rated as 7, 8, or 9, which were excluded from further analysis) (Figure 5). It was found that the cumulative contribution rate of the first principal component (PC1) was 87.5%, and the contribution rate of the second principal component (PC2) was 10.1%. PC1 showed a positive correlation with DKF, DKS, and ODK, while showing a negative correlation with CWC. PC2 showed a positive correlation with CWC and ODK, and a negative correlation with DKF and DKS. There was significant overlap between the samples of different maturities, and the samples with maturity 6 were generally clustered within the elliptical range of those with maturity 5. Therefore, it was concluded that when maturity 5 was reached, which corresponded to the sampling date of 14 October in most cultivars, the tea seeds were fully matured. The dry kernel content and ODK of tea fruits with the maturity of 5 were used as quantitative indicators, for directly calculating the tea seed oil output based on the fresh tea fruit yield in the actual production process and screening candidate cultivars for oil use. The calculated oil yield ranking across cultivars was as follows: HJJ > LJCY> ZY > HJY >YS > LJ43 > BY1 > ZC108 > YH1 > ZN117 >ZJ > SX > RX1> JX > JYMT. Based on this ranking, HJJ, LJCY, ZY, and HJY were selected as the most promising oil-use candidate cultivars for further evaluation and production.

### 3.5. Identification of Peaks in GC-MS Analysis of Tea Seed Oil Extracts

**Figure 6:**
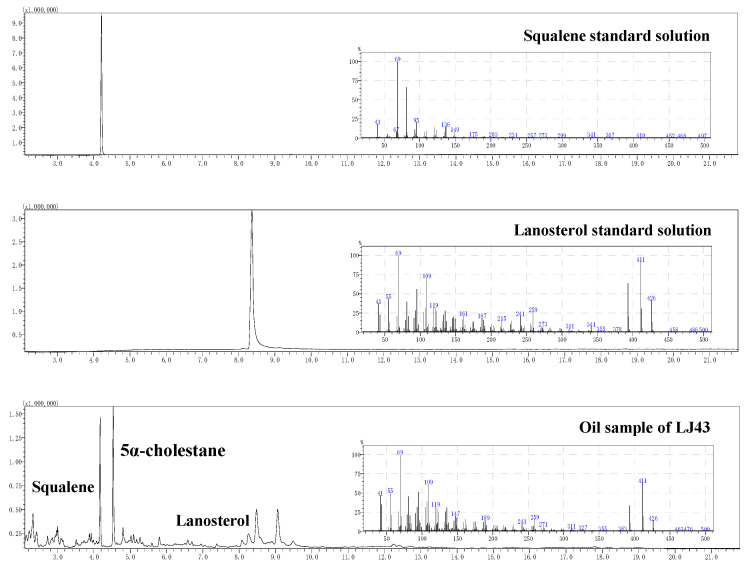
Total ion current and full scan mass spectrometry images of standard and extract of tea seed oil.

The squalene and lanosterol standard solution were analyzed following the tea seed oil extract detection protocol. The total ion chromatogram (Figure 6) demonstrated excellent separation of squalene and lanosterol, with the retention time of 4.215 min and 8.365 min, respectively. Using the n-hexane extract of LJ43 as the experimental sample, squalene, lanosterol, and 5α-cholestane were successfully identified through GC-MS NIST database matching and retention time verification, confirming good separation.

### 3.6. Squalene Content During Tea Seed Maturation

**Figure 7:**
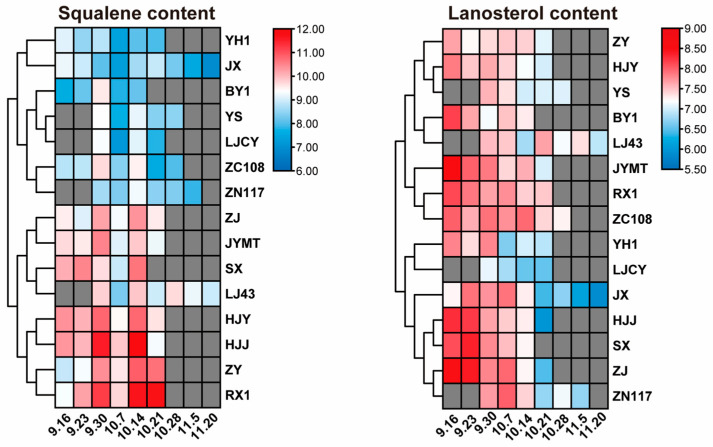
The heatmap of squalene and lanosterol content of tea seed oil from 15 different leaf-color tea cultivars. The contents were plotted on a log2 scale. The grey module indicated that the content of squalene in that day had not been detected.

To investigate the effect of maturity on the squalene content in tea seed oil, the squalene content in 90 tea seed oil samples was measured. Overall, tea cultivars with specific leaf colors exhibited higher squalene content at all stages compared to green (only LJ43 was relatively higher) (Figure 7). Among these, HJJ and RX1 displayed particularly high levels. Cluster analysis suggested that HJJ, RX1, ZY, and HJY were excellent candidate cultivars for high squalene content.

**Figure 8:**
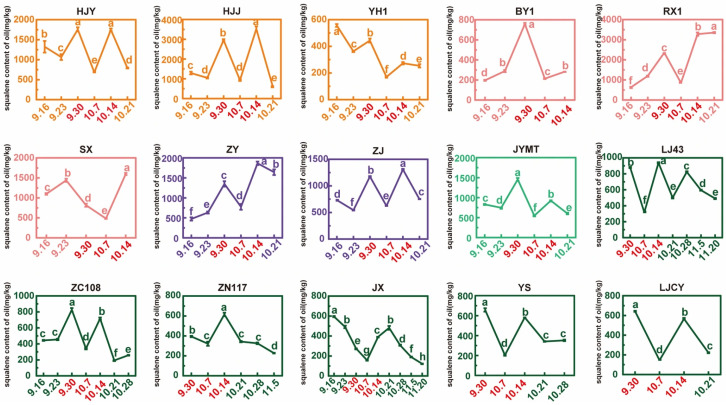
Changes in squalene content of oil from tea seeds in maturation process. The three dates marked in red indicated that the squalene content of samples collected on those specific days required particular attention. Different letters above bars indicated significant differences using Duncan’s tests (*p* < 0.05).

Comparative analysis of squalene content during maturation (Figure 8) revealed fluctuating trends among cultivars, contrasting with the unidirectional trends observed for other traits. HJY, HJJ, YH1, ZJ, and JYMT showed an initial decline followed by an increase from 16 September to 30 September. ZY, BY1, RX1, and ZC108 exhibited continuous increases. SX peaked then declined, while JX decreased steadily. From 30 September to 14 October, all cultivars displayed a sharp decline followed by rapid recovery. Seven cultivars (HJY, YH1, SX, JYMT, LJ43, YS, and LJCY) reached their lowest squalene levels on 7 October, while peak values predominantly occurred on 30 September or 14 October. For one group, BY1 (757.73 mg/kg), JYMT (1453.94 mg/kg), ZC108 (825.77 mg/kg), YS (657.18 mg/kg), and LJCY (642.04 mg/kg) showed the peak level on 30 September. For another group, HJJ (3524.36 mg/kg), ZJ (1308.78 mg/kg), ZY (1865.94 mg/kg), SX (1598.03 mg/kg), LJ43 (938.81 mg/kg), and ZN117 (615.50 mg/kg) showed the peak level on 14 October. YH1 (556.66 mg/kg) and JX (595.23 mg/kg) peaked on 16 September, while RX1 peaked on 21 October. Notably, when the highest value of cultivars was observed on 30 September, this was normally followed by the next highest values on 14 October. When the cultivars were observed with the highest values on 14 October, the second highest values for HJJ, ZJ, LJ43, and ZN117 occurred on 30 September. Additionally, the second highest value for YH1 was observed on 30 September, while the second highest value for RX1 occurred on 14 October. Finally, during the maturation process from 14 October to the following, the squalene content declined steadily across all cultivars. Combining the analysis of seed maturity for different tea cultivars in Section 3.4, we supposed that before the tea seeds were fully mature, the squalene content of different cultivars fluctuated sharply, but gradually decreased once fully mature.

### 3.7. Lanosterol Content During Tea Seed Maturation

Comparing the lanosterol content across various cultivars, the highest values for 13 cultivars occurred at the starting point of harvest (SX and JX at the second sampling point). Among them, JYMT, ZJ, SX, and HJJ all exceeded 300 mg/kg, while LJ43, YS, and LJCY was below 200 mg/kg. The lowest values for 12 cultivars occurred at the endpoint of harvest (RX1, YH1, and YS in close proximity) (Figure 7). Among these, BY1, RX1, and ZC108 exceeded 150 mg/kg, while YH1 and JX was below 90 mg/kg. Overall, cultivars with specific leaf colors tended to have higher lanosterol content than green. JYMT, ZJ, SX, HJJ, and RX1 could be selected as excellent candidate cultivars for tea seed oil rich in lanosterol.

**Figure 9:**
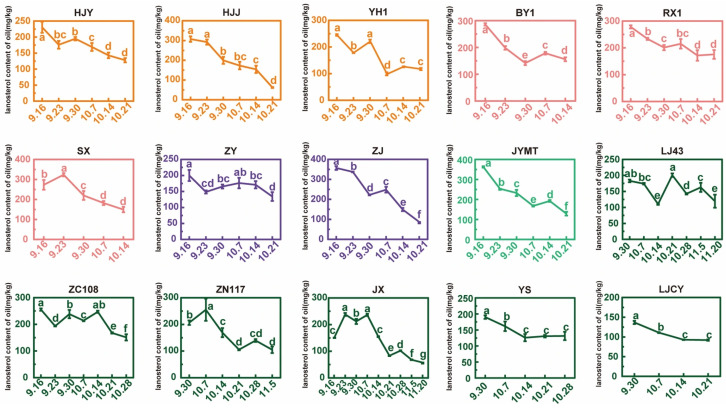
Changes in lanosterol content of oil from tea seeds in maturation process. Different letters above bars indicate significant differences using Duncan’s tests (*p* < 0.05).

Comparing changes in lanosterol content during maturation across the same cultivar, it fluctuated to some extent as maturity increased, but generally showed a decreasing trend (Figure 9). It was noteworthy that the magnitude of change during maturation differed among cultivars with different leaf colors. Some cultivars, such as HJY, ZY, and RX1, exhibited relatively stable changes, with overall variation around 100 mg/kg, while others, such as HJJ, ZJ, and JYMT, exhibited more drastic changes, with overall variation exceeding 200 mg/kg. The daily rate of decline at different maturity stages for the nine selected cultivars (as Section 3.1 mentioned) was calculated as 5.203 mg/kg, 2.373 mg/kg, 2.986 mg/kg, 3.000 mg/kg, and 6.757 mg/kg, with the highest rate of decline observed between 14 October and 21 October. In other words, after the tea seeds were fully mature, the content decreased particularly significantly.

## 4. Discussion

Most studies have primarily focused on the physiological and biochemical variations of oil-bearing crops such as *C. oleifera* and *Olea europaea* at different maturity stages [52,56,65,66]. There were relatively few reports in domestic and international research on the dynamic changes in seed traits during the maturation of tea (*C. sinensis*) seeds. This study aimed to extract high-yield and high-quality tea seed oil, identify the optimal harvest period for tea seeds with high levels of squalene and lanosterol, and select excellent cultivars with specific leaf colors for production. The study began at the stage when the seed kernel’s basic development transitions to a solid state, and ended during the fruit drop stage in the late maturation period. In numerous studies (including *C. oleifera*, *C. chekiangoleosa*, Japanese quince, peanuts, linseed, and almonds), the harvest dates have similarly been utilized to investigate fruit developmental processes and classify maturity stages [53,54,55,56,57,67]. In contrast, olive research has introduced a maturity index that integrates both harvest timing, seed skin, and pulp color [68]. Similarly, maturity assessment in bambara groundnut and jujube relied on pericarp characteristics [69,70]. In this study, we likewise accounted for pericarp morphological traits when sampling fruits across different dates to determine maturity progression.

### 4.1. Kernel Traits

Across all cultivars, seed and kernel diameter increased, and color darkened during maturation, which was consistent with the fruit development process of *C. oleifera* [57]. In previous studies, the average CWC of four tea cultivars (‘Huangdan’, ‘Rougui’, ‘Fuding Dabaicha’, and ‘Fuyun No. 6’) during their 50-day maturation process was 73.78% (18 September) and 54.90% (7 November), with a general trend of continuous decrease in all cultivars [45]. For ‘Tieguanyin’, the CWC decreased continuously with development, from 98.87% on 29 July to 59.13% on 18 November, with a daily decrease rate of 0.43% [55], which was consistent with the results of this study. In addition, the CWC of *C. oleifera* seeds exceeded 74.88% before July and then gradually decreased, reaching below 40% by 19 October [54]. Another study on *C. chekiangoleosa* reported that the CWC was 61.75–78.89% at the first harvest date (1 July) and 22.97–34.81% at the final harvest date (22 September), exhibiting a sharp decrease in the late stage [56]. These findings indicated that the variation in CWC during fruit maturation was generally similar across different species. The CWC of almonds during the late stage (24 August to 26 September/2 October ) decreased sharply comparing the first stage (2 August to 24 August) [57]. However, in *C. oleifera*, CWC exhibited a more rapid decline during the early maturation phase (28 July to 28 August), whereas no significant variation was observed in the late maturation stage (21 September to 19 October) [54]. The rate of CWC variation may be closely associated with sampling timing, fruit developmental stages, and even environmental factors.

During the maturation of tea seeds, a decrease in CWC is accompanied by an increase in dry matter content. In this study, both DKF and DKS exhibited an increasing trend in different cultivars. Production practices indicated that the yield rate and hundred-seed weight both contributed to assessing the developmental value and feasibility of seeds [71]. The average grain weight of *C. chekiangoleosa* seeds showed an upward trend, indicating that the dry matter in seed kernel was accumulating continuously during the development [56]. The same trend of variation also occurred in the weight of seeds and kernels in hemp, while no significant weight increment was observed during the late maturation phase, as well as the seed dry weight of camelina [72,73].

During seed development, the ODK generally showed an inverse relationship with the water content, too. It was reported that the ODK steadily increased from around 20% (1 September) to 35.19% (19 October) in previous study [74]. Later studies extended the time range of investigation, measuring from 30 June to 1 November. The oil content followed a ‘slow–fast–slow’ accumulation pattern, increasing steadily. The oil content increased rapidly in July and August, while the ODK was very low due to the liquid state of kernels. In contrast, from September to November, after seed hardening, the oil content increased from 8.69% to 18.70% [75]. Similarly, during the 50-day maturation process (from 18 September to 7 November), the average ODK of four cultivars increased rapidly from 11.63% to 24.24% [45]. Another study showed that the ODK of ‘Tieguanyin’ increased from 2.50% (29 July ) to 18.78% (18 November) [76]. In the ‘Changlin40’ cultivar of *C. oleifera*, the ODK gradually increased from approximately 40% (7 September) to 55.31% (19 October), and then remained stable [54]. In *C. chekiangoleosa* seeds, the oil content increased from 5.36% (1 July) to 64.13–67.28% (22 September) [56]. The ODK of Japanese quince increased from 1.4–1.8% to 10.4–12.6%, and did not differ at 2 weeks before and at full fruit maturity [55]. During the ripening process of *Acer truncatum* fruits, the oil content showed a trend of increasing initially and then slightly decreasing before stabilizing [77]. Studies have discovered that lipid degraded possibly during the late development of fruits [78]. Overall, oilseed crops typically had relatively high oil contents. In addition to maturity, factors such as year, cultivar, and geographical origin also influenced the oil content. The ODK from 45 cultivars collected from nine provinces over two consecutive years was 16.29–33.80%, showing significant differences [79]. Under the influence of various factors, the oil content ranges of tea plants reported in the above studies exhibited little variation with similar trend and were generally consistent with the findings of the present study, fully demonstrating the reliability of using oil content to indicate changes in tea seed maturity.

Under natural pollination, tea plants exhibited low fruit set rates, typically about 1% [80]. A survey of 351 tea germplasms classified fruit yield (750–4500 kg/hm^2^) into sterility, low-, medium-, and high-yield types [81], highlighting the impact of fertility on tea seed utilization. In addition, implementing scientifically sound field management practices, such as pruning and mulching with camellia shells and weedproof film, demonstrably enhanced both fruit quality and yield [82]. Thus, beyond the quantitative metrics (DKF, DKS, and ODK) proposed, future studies should integrate fertility as a critical criterion for oil-use cultivars screening. Cultivar, the cultivation and management measures, tree age, and insect pests all impacted the fruiting ability significantly [83]. Research reports indicated that ZY had strong fruiting strength, whereas HJJ and HJY also exhibit relatively high fruiting strength (field observations). In summary, to improve the yield of oil-producing candidate varieties, the field management strategies should be adjusted according to specific production objectives.

### 4.2. Squalene

In previous studies, squalene content from different provinces and cultivars across China was 183.19–872 mg/kg [41,42,43]. In tea seed oils from the Fujian region, including cultivars like ‘Huangdan’, ‘Rougui’, ‘Fuding Dabaicha’, and ‘Fuyun No. 6’, the average squalene content was 123.31–161.42 mg/kg [45]. In 69 tea seed oils from Jiangxi, the average squalene content was 110 mg/kg [44]. Another study has reported squalene content from 80 different cultivars nationwide, ranging from 34.39 mg/kg to 1724.63 mg/kg [40]. In the present study, the squalene content of 15 different leaf-color cultivars was 273.20–3524.36 mg/kg, which was relatively higher than the above studies. Above all, the squalene content varied among different cultivars of the same species. In olive oil, research also suggested that the squalene content was hereditary and could be used to distinguish different cultivars [84]. Moreover, squalene content exhibited differences across species. The squalene content in pumpkin, *Amaranthus*, olive, peanut, *C. oleifera*, rambutan, *K. coccinea*, linseed, and peony seed oil was 5906.9 mg/kg, 42,000 mg/kg, 6268.65–9526.97 mg/kg, 3467.4 mg/kg, 122.02–248.24 mg/kg, 21.48 mg/kg, 14.4–15.1 mg/kg, 2.9–5.7 mg/kg, and 26.58–55.72 mg/kg, respectively [22,25,32,33,35,53,54,58,85]. Overall, the squalene content in tea plants was relatively high, demonstrating its further potential for development and utilization.

From 18 September to 7 November with collecting samples every 10 days, Zheng et al. found that the squalene content exhibited two peaks during the maturation process, occurring on 28 September and 18 October, with a trough on 8 October. The squalene content also showed a ‘bimodal’ fluctuation [45], similar to our study results. Another related study on 13 tea cultivars, including YS, ZC108, and LJ43 [40], was consistent with the materials used in our study. From 18 August to 9 October, the squalene content fluctuated somewhat in the early maturation stages but generally showed a downward trend, similar with the present results. However, since the sampling did not continue, it was not possible to determine if the ‘bimodal’ fluctuation trend occurred during the later stages of maturation that year [40]. The variation in squalene content followed different patterns across different species, and even among different cultivars within the same species. Some studies also have found that the squalene content gradually increased before the fruit was fully mature, reached a peak at full maturity, and then gradually decreased. For example, the squalene content in *C. oleifera* oil increased sharply and reached 161.25 mg/kg on 28 September, followed by a rapid decrease in October [57]. In most studies on olive oil, the squalene content was found to decrease gradually with increased maturity [58,61,86,87]. A similar trend was observed for linseed oil [53]. However, in olive fruits from the Koroneiki cultivar (*O. europaea* L.) collected in November, December, and January, a gradual increase in squalene content with maturity was observed [88]. In another study of four major olive cultivars grown in northwestern China, it was found that the trend of squalene content also depended on the cultivar, with some cultivars showing little change in response to maturity [65]. The presence of squalene in the seed oil of all three genotypes of Japanese quince was only detected in the last month of fruit development, with no significant relationship found between variety, maturity, and content [55]. In the later stages of growth of *Torreya grandis*, the squalene content in the seed oil first decreased continuously, from 163.1 mg/kg (17 July) to 33.6 mg/kg (25 August), and rose again to 45.3 mg/kg (23 September) when nearing full maturity [89].

Above all, to obtain high squalene content in tea seed oil, the optimal harvesting periods were around 30 September and 14 October in that year. During the period when tea seeds were close to full maturity, the squalene content fluctuated sharply, which not only directly affected squalene accumulation but also presented significant challenges for the production of tea seed oil with high squalene content. Due to the effects of cultivar, climate, cultivation management, and other factors, the optimal harvesting periods may vary in different regions and years. Therefore, determining the optimal harvesting period based solely on time carries significant uncertainty. Further research was needed to explore the influence of environmental factors on squalene content, so that better guiding production practices based on the tea seed’s developmental and maturation stages.

The selection and adjustment of cultivars and optimal harvesting periods were not the only ways to optimize tea seed oil quality. Differences in processing methods also affect the squalene content [90,91], numerous studies have concentrated on refining and innovating the production processes, which represent another crucial avenue for quality improvement. One study compared the effects of conventional extraction methods (cold pressing, hot pressing, Soxhlet extraction) with novel techniques such as microwave-assisted extraction on the bioactive components of *C. oleifera* oil. The results indicated that both cold pressing and microwave-assisted extraction methods yielded high-quality oil with a significant concentration of bioactive substances [92]. In another comparative study, the chemical compositions of *Macadamia integrifolia* oils extracted through different methods—squeezing, solvent extraction, and aqueous extraction—were analyzed, revealing that squeezing resulted in the highest squalene content (261.88 mg/kg) [93]. A further study evaluated the extraction efficiency of squalene from *Bischofia polycarpa* seed oils using various solvents, including petroleum ether, n-hexane, ethyl acetate, Folch (chloroform/methanol, 2:1 *v*/*v*), Hx: Iso (n-hexane/isopropanol, 3:2 *v*/*v*), acetone, and isopropanol, finding that petroleum ether was the most effective solvent, yielding 55.21 mg/kg of squalene [94]. At present, petroleum ether was used for extraction of oil containing squalene from tea seeds.

### 4.3. Lanosterol

The differences of lanosterol content were possibly due to differences in the origin of materials, cultivars, maturity, processing methods, and measurement techniques. Lanosterol has also been detected in certain refined plant oils, such as refined olive oil (176.14 mg/kg), rice bran oil (89.16 mg/kg), rice oil (318.63 mg/kg), peanut oil (13.26 mg/kg), sesame oil (109.15 mg/kg), soybean oil (120.32 mg/kg), and canola oil (55.24 mg/kg), while it was not detected in palm oil, sunflower seed oil, or corn oil [21]. This indicated that the type of material directly affected the presence and concentration of lanosterol, suggesting that lanosterol could be considered a characteristic component of plant oils. In comparison, tea seed oil contained relatively higher lanosterol content. Previous studies have reported that lanosterol content from different tea cultivars was 1019.67–1680.95 mg/kg [41,43]. These values were higher than those measured in this study (93.15–247.17 mg/kg). Lanosterol content from different regions of China ranged from 1024.39 mg/kg to 3184.59 mg/kg, with significant differences [42]. Among 80 cultivars of tea seed oil from across the China, lanosterol content was 88.22–1953.83 mg/kg. The farther the geographical distance of planting, the greater the difference in lanosterol content of tea seed oil produced by the same cultivar [40]. Lanosterol content exhibited significant regional variability across studies, closely linked to growth environments, genetic backgrounds, and sampling representativeness. However, no lanosterol was detected in *C. oleifera* seed oils from regions such as Jiangxi and Hunan. The bound lanosterol content decreased to some extent during the deacidification of crude *C. oleifera* seed oil, and after 12 months of storage, the content decreased by 7.8% [95]. Moreover, different oil extraction methods, such as cold pressing or supercritical fluid extraction, result in significant variations [96]. In this experiment, the alkaline environment during the saponification process of tea seed oil extraction may have partially oxidized lanosterol, and during the distillation, some lanosterol may have also been separated into the saponifiable fraction, leading to a lower final result. Future research could include the measurement of components and content in the saponifiable fraction for improving the saponification process. Other studies have similarly found that lanosterol content in tea seed oil generally decreased with increasing maturity [40]. Lanosterol content in olive oil also exhibited a progressive decline with increasing fruit maturity [97], as well as total sterols [98].

Increasing the lanosterol content in tea seed oil was an important direction for optimizing its quality. Based on the above analysis, to obtain tea seed oil with high lanosterol content, it was advisable to harvest the tea seeds earlier. Harvesting around 30 September in that year could improve the quality of tea seed oil more effectively than harvesting around 14 October, as this was when the squalene content peaks, and lanosterol content remains relatively stable at a high level. Of course, the suitable time for picking still needed to be adjusted according to the actual situation of field production, and comprehensively considered in combination with the development and maturity period of the fruits.

Above all, both squalene and lanosterol are key intermediates in the terpenoid metabolic pathway, with lanosterol being a downstream product of squalene [99]. The present study found that most leaf-color specific tea cultivars contained higher levels of squalene and lanosterol. It was hypothesized that the more vigorous carotenoid metabolism in leaf-color specific cultivars may lead to the accumulation of more precursor substances, thereby providing a material basis for the substantial synthesis of squalene and lanosterol [100]. In addition to genetic factors, the fluctuating changes in squalene and lanosterol contents during tea seed maturation were also closely related to developmental stages and environmental regulation. Lanosterol content was higher in the early stage of maturation, whereas squalene content peaked in the middle and late stages of maturation. These findings suggested that in the early maturation stage, more squalene may undergo further reactions to synthesize lanosterol, while the decrease in lanosterol content may result from the attenuation of upstream synthetic reactions and the enhancement of downstream metabolic reactions. Environmental factors such as temperature, light, and water stress could indirectly regulate the metabolic flux of squalene and lanosterol by affecting the enzyme activities of the mevalonate (MVA)/methylerythritol phosphate (MEP) pathways [101]. On 7 October, most tea seed cultivars showed the lowest squalene content, which appeared to be unrelated to maturity. This low point might be linked to local climate or short-term weather conditions, as there had been persistent rain for several days before sampling, with heavy rainfall and relatively low temperature on the sampling day. Previous studies have also found that the accumulation of squalene and lanosterol was closely related to temperature [102]. Farnesyl pyrophosphate synthase (FPS) and squalene synthase (SQS) are key enzymes in the squalene metabolic pathway, and alterations in any of these steps can affect the accumulation of target substances. Overexpression of *PgFPS* in tobacco increased squalene content by 1.88 times [99]. The varied activity of SQS leads to diverse squalene content [103]. Therefore, the regulatory mechanisms of these key enzymes among different tea cultivars under variable environments remain to be further investigated.

## 5. Conclusions

The study found a close relationship between the crude water content, dry kernel content, and oil content of tea seeds and their maturity. Higher maturity resulted in lower crude water content and higher dry kernel and oil content. These traits effectively reflected the changes in tea seed maturity. During the maturation process, the lanosterol content showed an overall decreasing trend. However, the variation in squalene content was more complex. Before the tea seeds were fully mature, the squalene content of different cultivars fluctuated sharply, but gradually decreased once fully mature. At full maturity, cultivars with specific leaf colors typically had higher squalene content than green-leaf cultivars. In summary, ZY was an excellent cultivar for high tea seed oil yield and rich in both squalene and lanosterol, while HJJ and HJY were excellent cultivars for high tea seed oil yield and rich in squalene. And RX1 was an excellent cultivar for tea seed oil rich in both squalene and lanosterol. This study provides a theoretical basis for the comprehensive development and utilization of leaf-color specific tea cultivars in terms of oil, squalene, and lanosterol, and offers references for the selection of appropriate tea fruit harvesting periods based on different production objectives. However, due to the limitations in harvesting periods, tested cultivars, research years, and geographical regions, it is necessary to expand the experimental samples and comprehensively consider factors such as maturity, cultivars, and environment to obtain more generalizable conclusions. Based on the identified materials with high squalene and lanosterol contents, in-depth studies can be conducted on the molecular mechanisms underlying their accumulation, and key genes can be explored to provide directions for molecular selection breeding.

## Figures and Tables

**Table 1:** Yield of fresh and dry kernels in fresh fruits and seeds harvested in maturation process of HJY (%).

Date	KF	DKF	KS	DKS
16 September	36.37 ± 1.35 ^a^	9.83 ± 0.36 ^c^	61.50 ± 7.90 ^a^	16.62 ± 2.13 ^e^
23 September	33.19 ± 0.90 ^a^	10.36 ± 0.28 ^c^	64.48 ± 4.00 ^a^	20.13 ± 1.25 ^d^
30 September	36.14 ± 1.18 ^a^	14.24 ± 0.46 ^b^	67.19 ± 1.50 ^a^	26.47 ± 0.59 ^c^
7 October	32.91 ± 1.55 ^a^	14.42 ± 0.68 ^b^	62.54 ± 1.89 ^a^	27.40 ± 0.83 ^bc^
14 October	34.23 ± 2.65 ^a^	15.47 ± 1.20 ^ab^	65.11 ± 4.87 ^a^	29.41 ± 2.20 ^ab^

Note: The results were presented as mean ± standard deviation. KF, fresh kernel yield of fresh fruit (%); KS, fresh kernel yield of fresh seed (%); DKF, dry kernel yield of fresh fruit (%); DKS, dry kernel yield of fresh seed (%). Different letters above bars indicated significant differences using Duncan’s tests (*p* < 0.05).

**Table 2:** Correlation analysis of assumed maturity levels of different cultivars with four seed traits.

Cultivars	CWC	DKF	DKS	ODK
HJY	−0.949 **	0.931 **	0.927 **	0.979 **
HJJ	−0.981 **	0.976 **	0.988 **	0.869 **
YH1	−0.926 **	0.967 **	0.932 **	0.892 **
ZJ	−0.886 **	0.853 **	0.874 **	0.964 **
ZY	−0.974 **	0.949 **	0.967 **	0.796 **
BY1	−0.955 **	0.825 **	0.886 **	0.768 **
RX1	−0.937 **	0.801 **	0.844 **	0.930 **
SX	−0.972 **	0.965 **	0.977 **	0.892 **
JYMT	−0.944 **	0.848 **	0.932 **	0.918 **
LJ43	−0.980 **	0.942 **	0.973 **	0.586 **
ZC108	−0.937 **	0.914 **	0.923 **	0.965 **
ZN117	−0.527 *	0.521 *	0.589 *	0.778 **
JX	−0.944 **	0.961 **	0.960 **	0.938 **
YS	−0.861 **	0.800 **	0.857 **	0.705 **
LJCY	−0.766 **	0.749 **	0.797 **	0.866 **

Note: * and ** indicated significant difference at *p* < 0.05 and *p* < 0.01 level, respectively. CWC, crude water content of kernels (%); DKF, dry kernel yield of fresh fruit (%); DKS, dry kernel yield of fresh seed (%); ODK, oil content of dry kernels (%).

## Data Availability

The original contributions presented in this study are included in the article. Further inquiries can be directed to the corresponding author.
